# Social inequality and change in psychological distress during the Covid-19 pandemic in Norway

**DOI:** 10.1093/eurpub/ckac131.490

**Published:** 2022-10-25

**Authors:** BEV Aasan, M Lillefjell, K Kvaløy, S Krokstad, ER Sund

**Affiliations:** 1 Department of Public Health and Nursing, Norwegian University of Science and Technology, Trondheim, Norway; 2 Levanger Hospital, Nord-Trøndelag Hospital Trust, Levanger, Norway; 3 Department of Neuromedicine and Movement Science, Norwegian University of Science and Technology, Trondheim, Norway; 4 Department of Public Health, University of Stavanger, Stavanger, Norway; 5 Faculty of Nursing and Health Sciences, Nord University, Levanger, Norway

## Abstract

**Background:**

Emerging research findings suggest that the Covid-19 pandemic may have affected various social groups differently. Using follow-up health data from Norway, the aim of this study was to investigate whether change in psychological distress in adolescents during the Covid-19 pandemic differed across socioeconomic position (SEP).

**Methods:**

Data consisted of 1254 Norwegian adolescents who participated in Young-HUNT4 (2017-19) and Young-HUNT COVID (spring 2021). Psychological distress (PD) was assessed by the Symptom Checklist-10, using a cut-off point of ≥ 1.85 to identify adolescents with high PD. Based on the adolescents PD score prior to and during the Covid-19 pandemic, four groups were identified: persistent low PD, improved PD, worsened PD, and persistent high PD. Adolescents reported their parents’ education level which was used as an indicator for SEP. SEP was dichotomized separating low and high SEP, where having at least one parent with a higher education was defined as high SEP. Multinomial logistic regression models were used to investigate if the likelihood of the outcome groups varied across SEP, adjusted for age and sex.

**Results:**

Compared to the persistent low PD group, no statistically significant difference was found between low and high SEP in the improved PD and worsened PD group (RR 1.33 95% CI 0.77 - 2.31, RR 1.22 95% CI 0.82 - 1.81, respectively). However, adolescents of low SEP had a higher likelihood to be in the persistent high PD group compared with the high SEP group (RR 1.93 95% CI 1.30 - 2.87).

**Conclusions:**

These preliminary findings suggest that the pandemic may not have led to greater social inequality in mental health among Norwegian adolescents. However, the results showed that social inequality that existed prior to the pandemic has persisted, as adolescents of lower SEP had a higher likelihood of reporting high PD that persisted from prior to and throughout the Covid-19 pandemic.

**Key messages:**

• Social inequality in psychological distress among Norwegian adolescents has persisted prior to and during the Covid-19 pandemic.

• The Covid-19 pandemic does not seem to have increased existing social inequalities in psychological distress among Norwegian adolescents.

